# Severe acute maternal morbidity (SAMM) in postpartum period requiring tertiary Hospital care

**Published:** 2012-03

**Authors:** Seema Bibi, Saima Ghaffar, Shazia Memon, Shaneela Memon

**Affiliations:** 1 *Department of Obstetrics and Gynecology, Liaquat University of Medical and Health Sciences, Jamshoro, Pakistan.*; 2 *Countess of Lady Dufferen Fund (C.D.F), Maternity Hospital, Hyderabad, Pakistan.*; 3 *Department of Pediatric Medicine, Liaquat University of Medical and Health Sciences, Jamshoro, Pakistan.*

**Keywords:** *Severe acute maternal morbidity (SAMM)*, *Postpartum period*, *Tertiary hospital admission*

## Abstract

**Background: **Postpartum period is the critically important part of obstetric care but most neglected period for majority of Pakistani women. Only life threatening complications compel them to seek for tertiary hospital care. We describe the nature of these obstetric morbidities in order to help policymakers in improving prevailing situation.

**Objective**: To find out the frequency and causes of severe post-partum maternal morbidity requiring tertiary hospital care and to identify the demographic and obstetrical risk factors and adverse fetal outcome in women suffering from obstetric morbidities.

**Materials and Methods: **This prospective cross-sectional study was carried out in the Department of Gynecology and Obstetrics, Liaquat University Hospital Hyderabad, between April 2008-July 2009. The subjects comprised of all those women who required admission and treatment for various obstetrical reasons during their postpartum period. Women admitted for non-obstetrical reasons were excluded. A structured proforma was used to collect data including demographics, clinical diagnosis, obstetrical history and feto-maternal outcome of index pregnancy, which was then entered and analyzed with SPSS version 11.

**Results**: The frequency of severe postpartum maternal morbidity requiring tertiary hospital care was 4% (125/3292 obstetrical admissions). The majority of them were young, illiterate, multiparous and half of them were referred from rural areas. Nearly two third of the study population had antenatal visits from health care providers and delivered vaginally at hospital facility by skilled birth attendants. The most common conditions responsible for life threatening complications were postpartum hemorrhage (PPH) (50%), preeclampsia and eclampsia (30%) and puerperal pyrexia 14%. Anemia was associated problem in 100% of cases. Perinatal death rate was 27.2% (34) and maternal mortality rate was 4.8%.

**Conclusion**: PPH, Preeclampsia, sepsis and anemia were important causes of maternal ill health in our population. Perinatal mortality was high.

## Introduction

In South-east Asian and African countries, 8% of the global burden of the disease in women of reproductive age group is attributed to pregnancy and childbirth related conditions (1). Each year pregnancy related complications kills 536,000 mothers, destroy the health of 20 million women and also contribute to 2.5 million perinatal deaths (2, 3). 

Despite the fact, that majority of maternal deaths and disabilities develop in the postpartum period (4); postpartum care remains the most neglected aspect of obstetric services (5). Similarly in Pakistan, only 23% of women visit health care providers for postnatal consultations (6). As a result, a significant number of maternal morbidities in the puerperium remain undiagnosed, neglected and mismanaged. Poor women either die or suffer from chronic lifelong and debilitating complications like fistulas, pelvic inflammatory disease (PID) and infertility. 

Measuring maternal morbidity is a challenging job, owing to the absence of standard criteria and lack of population based data. In Pakistan, women usually do not attend the hospitals for routine postnatal consultations, only serious and fatal complications pursue them to reach the tertiary hospitals. 

Therefore tertiary care hospital data can be used as a proxy for moderate to severe morbidity in population. Referral to tertiary hospital is described as one of the criteria for measuring maternal morbidity in low income countries (7). 

The objective of the study is to find out the nature and risk factors of obstetric morbidities in special context to post partum period in order to enable health care providers and policy makers in improving the existing safe motherhood services in Pakistan and achieving the millennium development goal.

## Materials and methods

This department approved, prospective cross sectional study was carried between April 2008 to July 2009 in the Department of Gynaecology and Obstetrics Unit 3, Liaquat University Hospital (LUH) Hyderabad, Pakistan.

LUH is 1300 bed tertiary referral public sector hospital which serves a large number of mostly underprivileged populations of both urban and rural areas of lower Sindh. The study comprised of all those women who required admission and treatment in the Department of Obstetrics and Gynecology for various obstetric reasons during post partum period. 

Women admitted for various non obstetrical reasons were excluded as they were routinely shifted to concerned medical and surgical wards after initial management. Sample size of 125 was used taking the frequency of severe maternal morbidity as 8.23% (8). Criteria for the selection of maternal morbidity were based on the combined approach including clinical signs, symptoms, medical and surgical intervention required. 

**Statistical analysis**

A structured Performa was used to gather the information after managing and stabilizing the patients and taking verbal informed consent. Data included clinical diagnosis, demographic features, obstetrical records of index pregnancy and their fetomaternal outcome. Data was entered and analyzed on SPSS version 11 and simple frequencies and percentages were drawn.

## Results

In total 3292 women got admission in Obstetrical Ward during the study period, out of which 125 women were admitted in the postpartum period, making the frequency of postpartum obstetric morbidity as 4%. Causes, demographic and obstetrical risk factors are shown in table I and table II. Overall 61 (48.8%) women required blood transfusion, out of which 16 (12.8%) needed massive blood transfusion of more than 3 units of blood. 

Mean Hospital stay was 4.35±2.21 days. Among the study population the maternal and perinatal mortality was 6 (4.8%) and 34 (27.2%) respectively.

**Table I:** Causes of postpartum maternal morbidity requiring hospital admission (n=125).

**Variable**		**Frequency**	**Percentage**
Postpartum hemorrhage:		62	49.6%
	Uterine atony	28	22.4%
	RPOC’s/ Retained placenta	26	20.8%
	Genital tract injury	08	6.4%
Pre-eclampsia/ eclampsia		38	30.4%
Puerperal pyrexia/ sepsis		18	14.4%
Infected cesarean/ episiotomy wound		07	5.6%
Associated problems			
	Anemia	125	100%
	HCV* carrier	06	4.8%
	UTI/RTI**	04	3.2%

* Hepatitis C carrier.

** Urinary and respiratory tract infection.

**Table II:** Demographic and obstetrical risk factors (n=125).

**Variable**		**Frequency**	**Percentage**
Age (years)			
	20- 35	119	95.2%
	35 and above	06	4.8%
Residence			
	Rural	74	59.2%
	Urban	51	40.8%
Education			
	Illiterate	91	72.8%
	Primary	20	16.0%
	Secondary and higher	14	11.2%
Parity			
	Primipara	58	46.4%
	2-4	35	28.0%
	More than 4	32	25.6%
Antenatal visit to HCP		87	69.6%
Place of delivery			
	Home	40	32.0%
	Private health facility	32	25.6%
	Public health facility	53	42.4%
Mode of delivery			
	Vaginal birth	94	75.2%
	Caesarean section	31	24.8%
Delivery conducted by			
	Untrained birth attendant	40	32.0%
	Midwife / nurse	04	3.2%
	Doctor	81	64.8%
Referral			
	Self	42	33.6%
	Private health facility	33	26.4%
	Public health facility	50	40.0%
Delivery admission interval			
	Less than 24 hours	71	56.8%
	More than 24 hours	54	43.2%

## Discussion

Severe acute maternal morbidity (SAMM) cases are those in which women suffered from life threatening complications and who survived by good fortune and good hospital care. There is no internationally accepted classification for quantifying SAMM, however various approaches have been proposed by researchers including definitions based on clinical signs and symptoms, organ system failure, management and combined approaches. Each has its own limitations and mostly required clinicians for making diagnosis. It seems that combined approaches used by Souza and colleagues and others yield more comprehensive definitions of the maternal morbidities (7, 9).

In our study, the frequency of obstetric morbidities was 4 per 100 deliveries comparable with the frequency of 3.3 per 100 deliveries reported from tertiary hospital of Delhi, India (10). However, systemic review on the incidence and prevalence of SAMM showed a wide range of 0.07 to 8.23, which might be attributed to the use of different criteria for the identification of problem (8). 

The common obstetric morbidities for which women seek tertiary hospital admission and treatment were postpartum hemorrhage (PPH), pre-eclampsia and sepsis in line with the study from University Hospital of Damascus, Syria (11). These three conditions were not only the important causes of severe maternal morbidity but also found to be the major killers of pregnant women in the studies published from our own institution, nationally and worldwide (6, 12).

PPH was the biggest contributor of maternal ill health in our study. The majority of it followed uterine atony, which is said to be an unpredictable cause of PPH. However multiparity and presence of anemia in 100% of cases were the important risk factors detected in the study. Nearly similar figures were reported from Naz and colleagues who found anemia in 90% of cases of primary PPH (13). Promotion of deliveries by skilled birth attendants, ensuring active management of third stage of labour along with provision of cheap uterotonics like misoprostol will likely improve the prevailing situation (14).

Pre-eclampsia and sepsis were other important reasons for maternal ill health in the postpartum period requiring tertiary hospital admission, in line with another published report from Liaquat University Hospital where both these conditions were not only responsible for critically ill obstetrics patients who subsequently required transfer to intensive care unit (15). Pre-eclampsia related morbidity and mortality can be lessened by advocating evidence based low cost interventions like identifying high risk women and use of aspirin and calcium. 

Likewise promotion and practice of clean child birth practices including clean hands, a clean birth surface and a clean cord cutting will likely to reduce the sepsis related morbidity (16). An alarming fact of the study was the presence of anemia in all the critically ill patients. We could not predict whether it was the cause or consequence of maternal ill health due to unbooked status of women and lack of information regarding their hemoglobin levels in pregnancy. 

However according to World Health Organization, 39.1% (29.6-49.5%) of pregnant women in Pakistan are anemic which predisposes them to suffer from adverse fetomaternal outcome like postpartum hemorrhage, sepsis, preterm labour and low fetal birth weight (17). 

Development of complications, particularly PPH, acts in a vicious circle and further decreases their hemoglobin concentration. High prevalence of anemia in Pakistani women of reproductive age group is evident to be secondary to illiteracy, poverty, malnutrition and chronic infections like malaria, multiparity, lack of antenatal care and improper use of hematinics were other important contributing factors. 

An interesting feature of the study was increased utilization of health care facilities as about 70% of the study population had at least one antenatal consultation from health care provider and were delivered at hospitals by doctors. Lack of expertise along with lack of emergency obstetric care services might compelled doctors to refer the patients to tertiary care hospitals. Positive feature is proper and timely referral of patients as nearly 60% reached our hospital within 24 hours of delivery thus leads to decrease case fatality ratio for postpartum morbidity i.e. 4.8%. Recently Khan and colleagues, while discussing maternal health issues and options in Pakistan, highlighted increased utilization of private health care facilities due to lack of poor quality of services in public run hospitals. Our study also supported this fact, as a significant numbers of women have received intrapartum care at private maternity homes (6). 

Worldwide, the major causes of perinatal mortality are prematurity, birth asphyxia and infections secondary to pregnancy and delivery related complications. Our study also underscores this association in the form of low mean birth weight (2.52±0.51 kg) and alarmingly high perinatal mortality rate of 27%. Stillbirth shares 24% of the perinatal death rate. Thus supporting mothers during pregnancy, labour and puerperium will not only save mothers but likely will slash deaths of newborns (18). The limitation of this study is being hospital based, nonetheless in the absence of robust population based figures; such reviews do provide the opportunity to identify the main causes and the magnitude of SAMM in Pakistani population.

PPH, pre-eclampsia, sepsis and anemia are the principal causes of maternal ill health in the postpartum period. Malfunctioning of health care system secondary to absence of emergency obstetric and neonatal care (EMONC) services and properly trained staff at primary and secondary care level were found to be the important perpetrator. 

Prevailing situation can be improved by designing and implementing evidence based and cost effective management guidelines, training of health care providers and provision of EMONC services at primary and secondary health levels.
